# The change in weight perception of weight status among the overweight: comparison of NHANES III (1988–1994) and 1999–2004 NHANES

**DOI:** 10.1186/1479-5868-5-9

**Published:** 2008-02-12

**Authors:** Wendy L Johnson-Taylor, Rachel A Fisher, Van S Hubbard, Pamela Starke-Reed, Paul S Eggers

**Affiliations:** 1 US Department of Health and Human Services, National Institutes of Health, Division of Nutrition Research Coordination, Bethesda, MD, USA; 2 US Department of Health and Human Services, National Institutes of Health, National Institute of Diabetes and Digestive and Kidney Diseases, Bethesda, MD, USA

## Abstract

**Objectives:**

This study seeks to determine whether perception of weight status among the overweight has changed with the increasing overweight/obesity prevalence.

**Methods:**

The perception of weight status was compared between overweight participants (BMI between 25.0–29.9 kg/m^2^) from NHANES III (1988–1994) and overweight participants from NHANES 1999–2004. Perception of weight status was assessed by asking participants to classify their weight as about the right weight, underweight or overweight. Comparisons were made across age groups, genders, race/ethnicities and various income levels.

**Results:**

Fewer overweight people during the NHANES 1999–2004 survey perceived themselves as overweight when compared to overweight people during the NHANES III survey. The change in distortion between the survey periods was greatest among persons with lower income, males and African-Americans.

**Conclusion:**

The increase in overweight/obesity between the survey years (NHANES III and NHANES 1999–2004 has been accompanied with fewer overweight people perceiving themselves as overweight.

## Background

**Figure 1:**
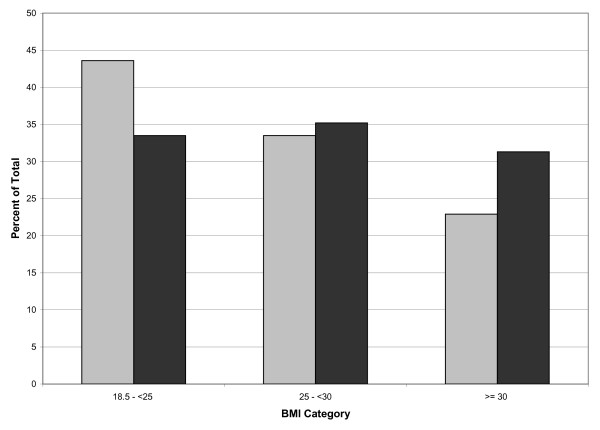
Comparisons of BMI categories between NHANES III (1988–1994) and NHANES 1999–2004, adults ages 20+. (light gray) – NHANES III (1988–1994). (black) – NHANES 1999–2004.

In 2001, *The Surgeon General's Call to Action to Prevent and Decrease Overweight and Obesity *was issued in response to the increasing population prevalence of overweight and obesity and the resulting public health threat [1]. At the time of this report, the prevalence of overweight (BMI 25 kg/m^2 ^– 29.9 kg/m^2^) and obesity (BMI ≥ 30 kg/m^2^) among adults was 34% and 30.5%, respectively, based on data from the National Health and Nutrition Examination Survey (NHANES) 1999–2000 [2] which reflected an increase from the 32% who were overweight and 22.5% who were obese during the NHANES III survey period (1988–1994) [3]. See Figure 1. The most recent overweight/obesity prevalence data from NHANES in 2003–2004 found that 34% of all adults ≥ 20 years of age were overweight and 32% were obese [2]. Clearly we are moving in the wrong direction. Though a number of different factors have been attributed to this occurrence, including fewer opportunities to be physically active and increased accessibility of food [1]; this study attempts to explore the potential contribution of alterations in self-perception of weight. For the purposes of this study, distorted weight perception occurs when one perceives his/her weight to be in a different category (underweight, overweight, just right) than would be determined when making a comparison to the NIH Clinical Guidelines on the Identification, Evaluation and Treatment of Overweight and Obesity in Adults – The Evidence Report [4].

Distorted weight perception is not a new phenomenon and is actually very well documented [5-16]. Studies in this area have consistently found that women (regardless of weight status category) are more likely to perceive themselves as overweight when compared to overweight men. Emslie et al found that among British bank and university workers, overweight women were more likely to perceive themselves as overweight than men [15]. This is also clearly illustrated in a study by Inoue et colleagues where they compared the agreement between measured BMI and perceived weight status. They found that 55.3% of normal weight women perceived themselves as overweight [16].

Caucasians are more likely to perceive themselves as overweight when compared to African Americans and Mexican Americans. Overweight people of higher socioeconomic status (SES) are more likely to perceive themselves overweight when compared to those of lower SES. Individuals with BMIs greater than 30 are more likely to perceive themselves overweight when compared to those with BMIs between 25.0–29.9 kg/m^2 ^[13]. This observation appears to hold true across all races and genders.

The overweight public's failure to accurately recognize their own overweight status prior to becoming obese may prevent them from changing behaviors that might contribute to additional weight gain. Therefore, it is important to understand the magnitude of weight status distortion within persons with BMI scores within the overweight range.

The intent of the present study was to determine if there is a trend for more individuals observed in the "overweight" BMI range to perceive themselves to be of "normal" or healthy weight by comparing NHANES (1999–2004) to NHANES III (1988–1994). This study also assessed whether income, race and gender moderated this effect.

## Methods

This study used data from NHANES III (1988–1994) and NHANES 1999–2004. NHANES is a multi-stage, stratified probability sample of non-institutionalized U.S. civilians, ages 2 months and older. The survey is conducted by the National Center for Health Statistics (NCHS), Centers for Disease Control and Prevention (CDC) and includes administration of questionnaires, a physical exam, and a battery of laboratory tests. Details of the questionnaires, the exam, and the tests have been described elsewhere [17-20].

### Analytic Sample

Adult participants (≥ 20 years of age) classified as overweight, identified by a measured BMI: 25.0–29.9 kg/m^2 ^(NHANES III, n = 5,653, NHANES 1999–2004, n = 4,645), were included in our sample. Respondents were excluded if they were pregnant or under the age of 20 (children/adolescents). Pregnancy status was determined using self-reported information as well as results of the urine test administered during the medical examination.

### Measures

***Height and weight ***were measured by trained examiners during the extensive medical evaluation at the Mobile Examination Center (MEC). Participants were weighed using a scale with a digital display readout, and height was measured using a stadiometer.

***Body Mass Index (BMI) ***was calculated based on these weight and height measurements and calculated as weight (kg)/height (meters) squared. Participants were then classified as obese (≥ 30.0 kg/m^2^), overweight (25.0–29.9 kg/m^2^), or normal weight (18.5 – 24.9 kg/m^2^), which is consistent with the NIH Clinical Guidelines on the Identification, Evaluation and Treatment of Overweight and Obesity in Adults – The Evidence Report [4].

***Weight perception ***was ascertained asking subjects during the face-to-face interview if they considered themselves to be overweight, underweight, or about the right weight. Less than 0.01% of respondents were missing weight status data, and these individuals were excluded from analyses examining weight distortion. At the time respondents were asked to report their weight status, they were also told that their weight would be measured at a follow-up session.

#### Poverty Income Ratio

Respondents were asked to select the category of income that most accurately reflected their total combined family income over the last 12 months. Reported income was then used to develop the poverty income ratio (PIR), which is the ratio of income to the family's appropriate poverty threshold. Using the PIR allows income data to be compared across various survey years. Income was categorized, as suggested by NCHS, using the PIR eligibility cut points for the United States Department of Agriculture (USDA) food assistance programs, into the following categories: low (0.000–1.850), middle (1.851–3.500), and high (3.501+) incomes [21]. Individuals with missing PIR data were excluded from all income analyses. This included 9.7% (551) overweight adults from NHANES III and 8.6% (399) overweight adults from NHANES 1999–2004.

#### Age

Non-age specific estimates were age-standardized to the 2000 Census using the standard population structure as suggested by NCHS. For specific estimates examining age, the following categories were used: 20–34, 35–49, 50–64, and 65+. These categories enable exploration of how weight perception differs across the age spectrum while providing a sufficient number of subjects in each group.

#### Race/Ethnicity

NHANES participants during both survey periods were asked to select the race and ethnicity that they most readily identified with. Responses were then systematically collapsed into the following categories of Non-Hispanic (NH) Whites, Non-Hispanic (NH) Blacks, Mexican-Americans and Other [21,22]. Only the first three categories are reported on in this paper.

### Statistical Analysis

Analysis of the NHANES data was conducted using SAS (version 9, SAS Institute Inc, Cary, NC) and SUDAAN (version 9, Research Triangle Institute, Research Triangle Park, NC) statistical software packages. To account for unequal probabilities of selection and non-response, all our analyses incorporated sample weights in order to provide national estimates of population proportions. Standard errors were calculated in SUDAAN based on the Taylor Series Linearization method [23]. Analyses were conducted separately for each survey period (NHANES III 1988–1994 and NHANES 1999–2004). The study population was stratified by gender, race, age and income. T-tests were used to assess differences between each of the individual groups and the reference population, which was defined as white males or white females, ages 20–34 or a PIR of 0–1.85. T-tests were also used to test differences in proportions between the two survey periods. A p-value < 0.05 was considered statistically significant.

## Results

Sixty-two percent of measured overweight (BMI ≥ 25 kg/m^2 ^and < 30 kg/m^2^) respondents in NHANES 1999–2004 identified themselves as overweight when asked whether they considered themselves underweight, overweight or just the right weight compared to 68% who were asked the same question in NHANES III (1988–1994). In both surveys, the percentage of overweight females who accurately identified their weight status as overweight was greater than the percentage of overweight males who accurately identified themselves as overweight. This was consistent across all racial/ethnic and age groups. However, those in the youngest age group (ages 20–34) in both genders experienced statistically significantly more alteration in perception of weight status than the older groups between the survey years with 13% fewer males and females ages 20–34 accurately identifying themselves as overweight in the 1999–2004 survey compared with the NHANES III (1988–1994) survey. See Table 1.

**Table 1:** Percent of Overweight Adults Who Perceived Themselves to be Overweight

	**Males**		**Females**	
	% perceived overweight		% perceived overweight	

	**NH III, 1988–1994**	**NH 1999–2004**	**NH III, 1988–1994**	**NH 1999–2004**

All^I^	57.1	51.3^a^	84.1	77.8^a^
				
**Race/Ethnicity**^I^				
Non Hispanic Whites	**60.4**	**56.4**	**89.7**	**82.1**^a^
Non Hispanic Blacks	40.9*	30.7*^a^	72.4*	59.7*^a^
Mexican Americans	45.4*	43.1*	67.4*	69.9*
				
**Age**				
20–34	**60.15**	**47.3**^a^	**91.31**	**78.1**^a^
35–49	61.24	53.9*	90.26	82.8^a^
50–64	56.46	58.6*	83.02*	81.9
65+	43.93*	45.2^a^	60.52*	62.8*

* Indicates a statistically significant difference in perception from reference group (in bold)

a. Indicates a statistically significant difference in perception across survey periods, p < 0.05

I. Age-standardized

Among both genders, Non-Hispanic blacks were least likely to perceive themselves as overweight during the 1999–2004 NHANES survey. Less than one-third of NH black males considered themselves to be overweight in NHANES 1999–2004, compared to 56.4% of NH white males. Among females for the same survey period (NHANES 1999–2004) 59.7% of NH blacks had an accurate perception of their weight status whereas 82.1% of NH white females had an accurate perception. This represents a shift from the NHANES III (1988–1994) data, which when compared to the NHANES 1999–2004 data shows that fewer Non-Hispanic Blacks (males and females), fewer Non-Hispanic Whites (males and females) and fewer Mexican American males accurately perceive their weight status in the latter survey. It is also important to note that the weight perception of Mexican Americans of both genders and white males did not change significantly over the survey years. In addition among all ethnic groups, NH blacks experienced the greatest change in weight status perception between the survey periods. See Table 1.

As PIR increased, the trend was for more people to correctly perceive themselves as overweight. This trend was consistent across all racial ethnic groups. However, whites with lower and middle PIR and blacks with lower PIR realized the greatest growth in distortion between surveys with 8%, 9% and 18% (respectively) fewer persons accurately identifying themselves as overweight (p < 0.05). Mexican Americans with lower PIR were more likely than their NH black counterparts and less likely than NH whites of the same income level to perceive themselves overweight. However, there was an insignificant difference across survey years for the low income Mexican Americans. See Table 2.

**Table 2:** Age-Standardized Percent of Overweight Adults who Perceive Themselves to be Overweight by Race/Ethnicity and Income

	Non-Hispanic Whites		Non-Hispanic Blacks		Mexican Americans	
	
	**NH III 1988–1994**	**NH 1999–2004**	**NH III, 1988–1994**	**NH 1999–2004**	**NH III, 1988–1994**	**NH 1999–2004**
**PIR 0–1.85**	**67.9**	**60.2**^a^	**55.6**	**37.5**^a^	**48.7**	**48.1**
PIR 1.86–3.50	75.1*	66.2^a^	57.9	52.5*	62.9*	61.4*
PIR > 3.50	71.8	68.5*	59.3	52.6 *	70.7*	63.9*

* Indicates a statistically significant difference in perception from reference group, (PIR 0–185%), within each survey period, p < 0.05

a. Indicates a statistically significant difference in perception across survey periods, p < 0.05

## Discussion

The intent of the present study was to determine if the increasing population prevalence of overweight and obesity has been accompanied by a trend for fewer individuals categorized in the "overweight" BMI range to perceive themselves to be overweight when comparing NHANES (1999–2004) to NHANES III (1988–1994). The results of the study clearly show that fewer overweight people with BMIs within the overweight range identified themselves as overweight in the 1999–2004 NHANES Survey than did in the NHANES III survey. In order to gain a better understanding of the degree of distortion along the BMI continuum, an ad hoc analysis was conducted to examine the shift in weight perception among males and females with a BMI of 25.0–<27.5 compared to those with a BMI of 27.5–<30 between NHANES (1988–1994) III and NHANES 1999–2004. In both males and females, there was a statistically significant decrease in the number of overweight adults with an accurate weight perception among those with a BMI of 25.0–<27.5 (p = 0.004 and p < 001 respectively). While individuals in the higher BMI category (BMI 27.5–<30) experienced a decrease in accurate weight perception, this difference was not statistically significant. Among the individuals with a BMI greater than 30, there was no change in perception across survey periods. The majority of males and females with a BMI above 30 are aware that they are overweight (87.0% and 95.5% respectively). The shift at lower BMI levels suggests the perception of a healthy weight is expanding to include those who are mildly overweight.

The findings of our study are consistent with those found in a 2006 Pew research survey where 9 in 10 Americans acknowledge that there is a weight problem in this country and 7 in 10 acknowledge that their family and friends have a problem, yet only 4 in 10 say they themselves are overweight. Within the same survey 51% of respondents whose reports of their own height and weight would result in them being classified as overweight perceived themselves to be just right [24]. The findings suggest that while people seemingly understand the concept of overweight, the slide rule by which they conceptually estimate their own weight status is skewed. This observation may contribute to the continued increase in prevalence of obesity. More research is needed to help us understand not only the reasons for the distorted perception but also the potential impact.

## Conclusion

The results of this study speak to the need to increase the awareness of the public's perception of weight status. As the weight status perception becomes more distorted it lessens the likelihood that individuals will make the recommended behavioral changes necessary to realize the health benefits associated with even small weight loss.

## Competing interests

The author(s) declare that they have no competing interests.

## Authors' contributions

WJT conceived of the study, participated in the design of the study, the analysis, drafted the manuscript and incorporated other author comments. RF participated in the design of the study, the analysis, assisted with the draft of the manuscript. VH participated in the design of the design of the study. PSR and PE assisted with the draft of the manuscript. All authors read and approved the final manuscript.
